# Effect of body composition on bone mineral density in Moroccan patients with juvenile idiopathic arthritis

**DOI:** 10.11604/pamj.2014.17.115.1838

**Published:** 2014-02-18

**Authors:** Dalal El Badri, Samira Rostom, Ilham Bouaddi, Asmae Hassani, Bouchra Chkirate, Bouchra Amine, Najia Hajjaj-Hassouni

**Affiliations:** 1 Department of Rheumatology, EL Ayachi Hospital, University Hospital of Rabat-Sale, 11000, Sale, Morocco; 2 Department of Pediatrics, children’s Hospital, University Hospital of Rabat-Sale, 11000, Rabat, Morocco

**Keywords:** Body composition, lean mass, fat mass, juvenile idiopathic arthritis

## Abstract

**Introduction:**

The link between bone mass and body composition is widely recognized, but only few works were selectively performed on subjects with juvenile idiopathic arthritis. The aim of our study was to investigate the effect of body composition on bone mineral density (BMD) in Moroccan patients with juvenile idiopathic arthritis.

**Methods:**

Thirty three children with juvenile idiopathic arthritis (JIA) were included in a cross-sectional study. The diagnosis of JIA was made according to the criteria of the International League of Association of Rheumatology (ILAR). Body mass index (BMI) was calculated from the ratio of weight/height^2^(kg/m^2^). Pubertal status was determined according to the Tanner criteria. Bone status, body composition and bone mineral content (BMC) were analyzed by using dual-energy X-ray absorptiometry (DXA). BMD was assessed at the lumbar spine (L1-L4) and at total body in (g/cm^2^). Total body fat tissue mass (FTM) and lean tissue mass (LTM) were also analyzed by DXA and expressed in kilograms. In children, low BMD was defined as a Z-score less than -2 and osteoporosis was defined as a Z-score less than -2 with a fracture history.

**Results:**

A cross-sectional study was conducted in 33 Moroccan patients with JIA aged between 4 and 16 years, Fat mass was not related to bone density; in contrast, BMD was positively associated to LTM in total body(r = =0.41, p= 0.04) but not in lumbar spine (r = 0.29, p= 0.17). There exist significant correlation between BMC and BMD in total body (r = 0.51, p = 0.01).

**Conclusion:**

This study suggests that the LTM is a determining factor of the BMD during adolescence. Other studies with a broader sample would be useful to confirm this relation.

## Introduction

Childhood and adolescence are crucial periods for maximum bone mass acquisition, which is associated with genetic potential, nutritional factors, physical activity, and body composition [1]. Juvenile idiopathic arthritis (JIA) is comprised of a heterogeneous group of several disease subtypes that are characterized by the onset of arthritis prior to the age of 16 years; it’s one of the commonest rheumatic diseases of children and an important cause of short- and long-term disability [2–5]. Osteoporosis is commonly observed in children with JIA [6] who are prone to changes in body composition because of disease and treatment related factors [7]. Deficits of muscle mass have been described as a central factor in (secondary) bone loss [8, 9]. Apart from low bone mineral density (BMD) and lean mass, a higher fat mass was reported in children with rheumatic diseases [10]. In adults, this is summarized as rheumatoid cachexia [11]. Last years researches brought evidence for a strong relationship between body composition and bone mass [12]. However, association studies between body composition and BMD gave conflicting results, some reports showing a positive correlation between fat mass (FM) and BMD [13, 14], while no association [15] was obtained by others, only few works were selectively performed on subjects with JIA. The aim of our study was to evaluate the influence of body composition parameters on peak bone mass in a group of Moroccan children with JIA.

## Methods

### Patients

This cross- sectional study was conducted in 33 children and adolescents, aged between 6 and 16 years, with JIA, currently observed at the Children’s Hospital and department of Rheumatology of the University Hospital of Rabat-Sale Morocco. The diagnosis of JIA was made according to the criteria of International League of Associations for Rheumatology (ILAR) [16]. Parental consent was obtained from all participants. None of the subjects in this study had a medical history of chronic disease (endocrinal, neurological, cardiac, and renal) and no patient was taking bone sparing drugs.

The medical records of these patients were reviewed and information on the following parameters was extracted: age, sex, personal or family antecedent of fracture, subtype of JIA, disease duration, corticosteroid requirement (duration of corticosteroid use, cumulative corticosteroid dose calculated in mg as the product of mean daily dose × 365 × duration in years), requirement for and duration of other medications: methotrexate, salazopyrine and non-steroidal anti-inflammatory drugs (NSAIDs), Calcium and vitamin D supplementation. The functional disability was assessed by the Moroccan version of Childhood Health Assessment Questionnaire (CHAQ) [17], and the disease activity by the tender and swollen joint counts, patient assessment of pain and global disease activity, physician assessment of global disease activity, erythrocyte sedimentation rate (ESR), C-reactive protein (CRP), disease activity score (DAS 28) for polyarticular and oligoarticular JIA [18], the Maastricht AS Enthesitis Score and Bath AS Disease Activity Index (BASDAI) for juvenile spondylarthropathy [19].

Pubertal status (pre- or postpubertal) was determined using breast and pubic hair stages in girls, testicular and pubic hair stages in boys, according to the Tanner criteria [20]. The participant’s weight was measured in kg using a digital scale. Height was measured in meters using a vertical stadiometer. Based on these data, BMI (kg/m^2^) was calculated and it was also expressed as BMI-Z score, according to the World Health Organization Child Growth Standards (WHO) [21].

### BMD measurement

Bone mineral density (BMD), bone mineral content (DMC) and body composition were analyzed by a single physician using the same dual energy radiography absorptiometry (DEXA), using (Lunar Prodigy; GE Lunar, Madison, WI) equipped with standard density software. Total body fat mass and lean mass were analyzed by DXA and expressed in kilograms. Bone mineral density of the L1-L4 lumbar spine and total body was evaluated in grams/centimeter^2^ and expressed as Z score for age, sex and ethnicity according to the reference data given for this equipment.

According to the International Society for Clinical Densitometry recommendations osteoporosis was defined as a Z-score less than -2 with a fracture history. Low BMD was defined as a Z-score less than -2 without a significant fracture history and osteoporosis was defined as a Z-score less than -2 with a fracture history [22–23]. Medical history for bone fractures was negative in all patients. All subjects underwent plain Vertebral Fracture Assessment (VFA) [24] to exclude unknown vertebral fractures.

### Statistical analysis

Statistical analysis was performed using a software program (SPSS for Windows, Version 13.0, SPSS Inc, Chicago, IL). The data were expressed as the mean ± SD for continuous variables and as frequency (%) for categorical variables. Pearson or Spearman correlation coefficients were calculated to express association of continuous data with parametric or non-parametric distribution. One-way Anova was used to calculate differences between means. P values <0.05 were considered statistically significant.

## Results

### Demographic and disease characteristics

The study included 33 patients (15 females) with a median age of 11(5.75-14)years and median disease duration of 2(1-4.5) years. Twelve of the patients were at prepubertal and thirteen were at pubertal period. The medians for weight, height, and BMI were -1 (-2.05-0) SD and -1(-2.5-0) SD, 5(2-6) SD respectively. Obesity (BMI> +2.0) was detected in 5 (15.15%) of the patients, 22.2% (4/18) of the boys and 6.66%% (1/15) of the girls. The socio-demographic and clinical characteristics of the patients are presented in Table 1.

**Table 1:** Socio-demographic and clinical characteristics of the patients

Characteristics	
**Age (years)**^3^	11[5.75-14]
**Female sex**^1^	15(45.5)
**Tanner**^1^	
Prepubertal	12(36.4)
Pubertal	13 (39.4)
Postpubertal	8 (24.2)
**Weight/age**^1^**(Z)**	
-2DS	12(36.4)
Normal	20(60.6)
+2DS	1(3)
**Height/age (Z)**^1^	
-2DS	10(30.3)
Normal	23(69.7)
**BMI**^1^	
Obesity	5(15.2)
Normal	19(57.6)
Underweight	9(27.3)
**Subtype of AIJ**^1^	
Systemic	8(24.2)
Oligoarticular	9(27.3)
Polyarticular	16(48.5)
**Duration of disease, year (range)**^3^	2[1-4.5]
**DAS28**^2^	5.33 ± 1.11
**ACPA Positive**^1^	5(15.2)
**Rheumatoid factor (RF) Positive**^1^	4(12.1)
**Antinuclear antibodies positive**^1^	25 (76)
**ESR**^3^	35[25-50.5]
**CRP**^3^	20[10.5-40]
**Medications used**	
NSAID^1^	26 (79)
Corticosteroids^1^	19(58)
Cumulative dose of Corticosteroid^1^	10000(5470-21900)
DMARDs^1^	17(51.5)

1 Number and percentage

2 Mean and standard deviation

3 Median (quartile).

DAS: disease activity score, ACPA: Anti-citrullinated peptides antibodies ESR: Erythrocyte sedimentation rate; CRP: C-reactive protein; NSAIDs: non-steroidal anti-inflammatory drugs; DMARDs: disease modifying anti-rheumatic drugs

### Bone mineral density

**Figure 1:**
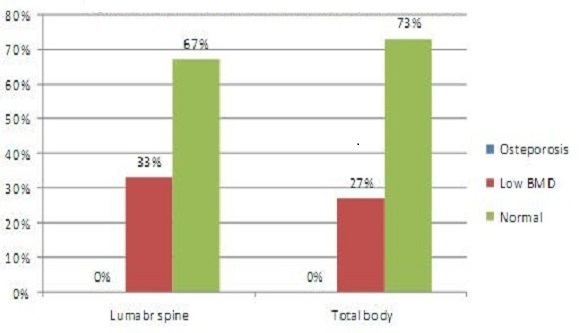
Bone mineral density on the patients with juvenile idiopathic arthritis in lumbar spine and total body

Eleven patients (33.3%) were given a diagnosis of low BMD in lumbar spine, and nine (27%) in total body (Z-score < -2), and no patient was given a diagnosis of osteoporosis (Z-score < -2 and a significant fracture history) (Figure 1). The median of lean tissue mass (LTM), total body fat tissue mass (FTM) and bone mineral content (BMC) were 19001g (13827-33140)4930 g (3385- 9139)and 1044.90 g (630.40-1808.90) respectively. The mean Z-score of obesity group was 0.08±0.93. The BMD and Z scores of patients according to BMI are presented in Table 2.

**Table 2:** Bone mineral density and Z scores of patients according to BMI

	Underweight	Normal	Obesity	P	
**lumbar spine**	BMD	0.665±0.20	0.639±0.24	0.876±0.15	0.13 (**0.009)**
	Z scores	-2.11±1.33	-1.58±1.20	0.08±0.93	
**Total body**	BMD	0.754±0.21	0.798±0.14	0.716±0.26	0.63(0.23)
	Z scores	-2.1±1.34	-1.41±0.85	-1.34±1.08	

BMD: Bone mineral density in (g/cm^2^); BMI: Body mass index

### Variables associated with parameters of body composition

LTM was correlated with age and Tanner stage. However we didn’t find any correlation between these parameters and FTM (Table 3). Bone mineral density in total body was positively associated to LM in our study (r = 0.41; p = 0.04). In contrast, FM was not related to BMD of the lumbar spine and total body (Table 3).

**Table 3:** Correlation between body composition and clinical characteristics of patients

	Lean mass		Fat mass		BMC	
	r	p	r	p	r	p
**Age**	0.58	**0.003**	0.24	0.26	0.78	**0.001**
**Tanner stage**	0.47	**0.02**	0.19	0.38	0.75	**0.001**
**Cumulative dose of Corticosteroid**	-0.12	0.71	0.24	0.47	-0.06	0.84
**BMI**	0.45	**0.03**	0.46	**0.02**	0.68	**0.001**

BMI: Body mass index BMC: Bone mineral content

**Table 4:** Relationships between body composition parameters and bone mineral density

	BMD in lumbar spine		BMD in total body	
	r	p	r	p
**Lean mass**	0.29	0.17	0.41	**0.04**
**Fat mass**	-0.22	0.30	-0.02	0.92
**BMC**	0.20	0.35	0.51	**0.01**

BMD: Bone mineral density; BMC: Bone mineral content

## Discussion

In this analysis of the relationship between body composition parameters and BMD in Moroccan patients with JIA, a positive association was observed between LM, BMC and BMD, and no correlation was found between FM and BMD consistent with previous studies suggesting that LM represents an important determinant of cortical bone mass accrual in childhood [25–27]. In a study on the relationship between lean and fat mass and bone mineral in young adults and adolescents, Janicka et al. found that lean mass, but not FM was associated with BMD, suggesting that muscle mass is the major body composition parameter stimulating bone mineral acquisition [28].

Previous studies indicated that regardless of age or gender, lean mass has a strong positive influence on BMD [29]. However, the results of previous studies on the relation between fat mass and BMD were conflicting. Adipose tissue can be a weaker positive predictor [29] or stronger predictor [30] than lean mass, or even a negative predictor of BMD [28].

Low fracture risk in obese people suggests a role of fat tissue in maintaining bone mass, either mediated by mechanical loading or due to bone-active adipokines such as leptin [12]. Leptin has been proposed to be a mediator of adipose tissue hormonal effect on bone mass [31]. The role of leptin in bone metabolism is not fully understood, but in animal studies, leptin deficient mice have demonstrated a high bone mass phenotype [32].

Studies on body composition in inflammatory disease are more frequent in RA. In general, these studies have shown greater lean mass loss, especially in those with higher activity of disease and disability [33]. In adult patients with rheumatic arthritis, the abnormalities of body composition have been termed as rheumatoid cachexia [34].

Tumor necrosis factor-α seems to be a central mediator of muscle wasting [35]. Low physical activity predisposes the body to fat gain and is believed to precipitate a negative reinforcing cycle of loss of muscle mass [36].

In children with JIA, especially in severe forms, smaller increases in lean mass, larger gain in fat mass and lower bone mineral content were found, compared with healthy controls [37–39].

In our study, we find that BMC increased with age, puberty stage, and BMI, De Schepper et al. in a study conducted in136 normal growing children between the ages of 1 and 18 yr found that BMC increased more rapidly during puberty. For the entire group, the increase in BMC with age, height, and weight was best predicted by an exponential regression line analysis [40].

To our knowledge, the present study is the first to report effect of body composition on bone mineral density in Moroccan children with JIA but it should be noted that the cross sectional nature of this study limits the interpretation of our results especially to infer causality. Another limitation is the luck of a group of healthy participants.

## Conclusion

In conclusion, in Moroccan patients with JIA, lean mass has a favorable effect on BMD. Fat mass seems not to protect the bone structure against osteoporosis. Further prospective studies with healthy participants are necessary to confirm our findings.
